# Hypoxia-conditioned media allows species-specific attraction of bone marrow stromal cells without need for recombinant proteins

**DOI:** 10.1186/1746-6148-10-56

**Published:** 2014-03-04

**Authors:** Anastasia Gabrielyan, Sven Knaak, Michael Gelinsky, Stefan Arnhold, Angela Rösen-Wolff

**Affiliations:** 1 Department of Paediatrics, University Clinic Carl Gustav Carus, TU Dresden, Dresden, Germany; 2 Centre for Translational Bone, Joint and Soft Tissue Research, University of Technology, Dresden, Germany; 3 Institute of Veterinary Anatomy, Justus-Liebig University, Giessen, Germany

**Keywords:** Stem cells, Migration, Hypoxia, Tissue repair

## Abstract

**Background:**

In vivo tissue regeneration depends on migration of stem cells into injured areas, their differentiation into specific cell types, and their interaction with other cells that are necessary to generate new tissue. Human mesenchymal stem cells, a subset of bone marrow stromal cells (BMSCs), can migrate and differentiate into osteoblasts in bone tissue. This can be facilitated by recombinant growth factors and cytokines. In many animal species, the availability of genomic sequences, recombinant proteins, and/or antibodies is limited so that new approaches are needed to generate resources that facilitate migration of stem cells into tissue defect areas. Here we used bone marrow stromal cells of human, ovine, equine, and canine origin to generate hypoxia-conditioned media (HCM) in order to attract BMSCs of the respective species in migration assays.

**Results:**

We show that HCM contain attractors even more potent than vascular endothelial growth factor and can therefore be used in many animal species without the need for purified proteins.

**Conclusion:**

Generation of HCM is easy and cheap compared to preparation and purification of protein fractions and/or recombinant proteins. Hence, HCM could be applied in large animals (e.g. sheep, horse, dogs) for attraction of BMSCs into tissue defects caused by tumor resection or trauma.

## Background

In vivo tissue regeneration depends on migration of stem cells into injured areas, their differentiation into specific cell types, and their interaction with other cells that are necessary to generate new tissue. Mesenchymal stem cells, a subset of BMSCs, can migrate and differentiate into osteoblasts in bone tissue [1,2]. Human BMSCs show significant chemotactic responses to several factors, including platelet-derived growth factor (PDGF), vascular endothelial growth factor (VEGF), insulin-like growth factor (IGF-1), interleukin-8 (IL-8), bone-morphogenetic protein (BMP)-4, and BMP-7 [1]. We have previously shown that human BMSCs can be attracted into three-dimensional scaffolds following a gradient of recombinant human stromal cell-derived factor 1α (SDF-1α) [3]. BMSCs themselves secrete significant levels of chemoattractive agents like VEGF, monocyte chemoattractant protein-1 (MCP-1), macrophage inflammatory protein-1α (MIP-1α), MIP-1β, and monokine induced by IFN-γ (MIG) [4]. Secretion of VEGF from these cells can be upregulated by hypoxic conditions in a hypoxia-inducible factor-1 (HIF-1)-dependent way [5].

Many chemoattractive agents of human origin are available as recombinant proteins that facilitate the study of migration processes in human cells. However, molecular biological characterization of BMSC migration in large animal species is difficult because of the limited availability of genomic sequences, recombinant proteins, and/or antibodies so that new experimental approaches are required. Here we used BMSCs of human, ovine, equine, and canine origin to generate hypoxia-conditioned media (HCM) in order to attract BMSCs of the respective species in migration assays. We show that HCM is an even more potent attractor than purified VEGF and can therefore be used in many animal species without the need for recombinant or otherwise purified proteins. We were able to show the presence of VEGF and high-mobility group protein B1 (HMGB1) in all HCM. HMGB1 is a chromatin-associated protein that binds to DNA and alters its conformation [6]. Released by hypoxic cells, it binds to the receptor for advanced glycation end-products (RAGE) and activates MAP kinase cascades [6]. It plays an important role in the process of BMSC migration [7].

## Methods

### Isolation and cultivation of human, ovine, equine, and canine BMSCs

Human BMSCs were obtained from the Translational Biomedical Research Group, Center for Regenerative Therapies, Dresden. Ovine bone marrow aspirates obtained from the chest of a merino sheep were provided by the Department of Orthopaedics, University Clinic Carl Gustav Carus, Dresden. Equine and canine BMSCs were isolated by the Department of Veterinary Anatomy, University of Giessen. Density gradient centrifugation by Ficoll (Biochrom, Berlin, Germany) was performed to enrich human, ovine, equine, and canine bone marrow stromal cells (BMSCs). Thereafter, they were cultured in T-175 flasks (Greiner Bio-One, Frickenhausen, Germany) in alpha medium (Biochrom) containing 10% fetal calf serum (FCS) (Sigma), 1% L-glutamine (PAA, Pasching, Austria) and 1% penicillin/streptomycin (PAA) in a humidified atmosphere with 20% O_2_, 5% CO_2_ at 37°C (Thermo Scientific BBD 6220 CO2 Incubator, Omnilab, Bremen, Germany). After 5 days the culture medium was exchanged and thereafter every 3-4 days until the culture reached 80-90% confluence.

### Cultivation of HUVECs

Human umbilical vein endothelial cells (HUVECs) were purchased from Promocell, Heidelberg, Germany and cultured in Endothelial Cell Growth Medium (ready-to-use, Promocell) without further supplements.

### Characterization of human, ovine, equine, and canine BMSCs

CD105 and CD271 MicroBead kits (Miltenyi Biotec) were used for magnetic labelling and positive selection of the BMSCs according to the manufacturer’s instructions.

### Generation of HCM

Human, ovine, equine, and canine BMSCs were cultured in T-175 flasks in alpha medium containing 10% fetal calf serum,1% L-glutamine and 1% penicillin/streptomycin (PAA) in a normoxic chamber until the cultures reached 80-90% confluence. After the passage, cells were cultured overnight in alpha medium containing 5% fetal calf serum,1% L-glutamine and 1% penicillin/streptomycin (PAA). On the next day, cultures were transferred into another incubator and cultured in a humidified atmosphere with 1% O2, 5% CO2 at 37°C for 48 h (Thermo Scientific HERAcell 150i, Waltham, MA, USA). Thereafter, the supernatants (HCM) were aliquoted into 2 ml tubes (Eppendorf) and stored at -80°C to be used in migration assays. Control media were generated in the same way but at normoxic conditions.

### Human, ovine, equine and canine VEGF and HMGB1 ELISA of HCM

In order to quantify the content of VEGF and HMGB1 in human, ovine, equine and canine HCM, an enzyme-linked immunosorbent assay (ELISA) was performed according to the manufacturer’s instruction. VEGF kits for human and canine ELISA were from R&D (Abingdon, England), for ovine from TSZ Biotang (Waltham, USA) and for equine from Genorise (Philadelphia, USA). HMGB1 kit was from IBL (Hamburg, Germany).

### Migration assay

Cell migration assays were performed in Corning Transwell®-96 permeable support chambers with porous polyester membranes with a pore size of 8.0 μm (Corning Incorporated Life Sciences, Munich, Germany). Human BMSCs (P2-11), ovine BMSCs (P5-P12), equine BMSCs (P4-7), and canine BMSCs (P5-8) were resuspended at 5 × 10^4^ cells/ml in Alpha Medium supplemented with 1% L-glutamine, 1% penicillin/streptomycin, without BSA and seeded into the upper chamber. HUVECs (P6) were resuspended in endothelial cell growth medium without supplements. 200 ng/ml human, ovine, equine and canine VEGF-A (all from Biomol, Hamburg, Germany) or HCM from corresponding MSCs were used as chemoattractive agents in the lower compartment. The 96-well plate was incubated overnight-night at 37°C in a normoxic incubator. The cells on the upper membrane compartment were removed manually. Then the migrated cells adhering to the underside of the membrane and from the lower chamber were dislodged by incubating the inserts in 0.25% trypsin for 4 min followed by treatment with trypsin neutralisation solution (Promocell). AlamarBlue® (Invitrogen, Darmstadt, Germany) was used to stain the cells overnight. Cell number was calculated at excitation 560 nm/emission 590 nm using Tecan microplate reader (Männedorf, Switzerland).

Results are described as the mean percentage of migrated cells over control cells, the latter show basal migration without chemotactic signal. Each condition was tested in four wells, experiments for human BMSCs and ovine BMSCs were repeated six times, for HUVEC, equine BMSCs and canine BMSCs three experiments were performed.

### Statistical analysis

T test was performed using the program available at http://www.daten-consult.de/forms/ttestunabh.html website.

## Results

### VEGF and HMGB1 ELISAs of human, ovine, equine, and canine HCM

**Figure 1:**
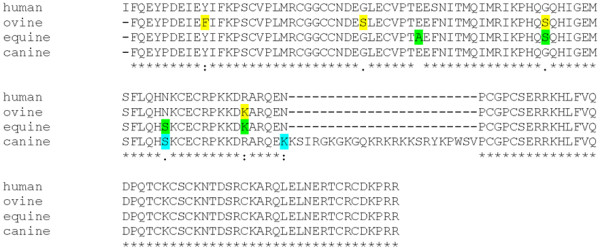
**Alignment of human [GenBank: AY047581.1], ovine [GenBank: AF071015.1], equine [NCBI Reference Sequence: NM_001081821.1] and canine [NCBI Reference Sequence: NM_001003175.2] VEGF amino acid sequences.** Differences to the human VEGF sequence are marked in yellow (ovine), green (equine), or blue (canine).

We performed ELISAs in order to determine the amount of VEGF and HMGB1 released from BMSCs under hypoxic conditions. When we started our experiments, only human VEGF kit was available; therefore, we tested if ovine, equine, or canine VEGF could be detected by antibodies directed against human VEGF by using human, ovine, equine, and canine HCM. As expected, human VEGF could be detected at a concentration of 1.99 ng/ml in human HCM. In canine HCM, we determined a concentration of 1.96 ng/ml VEGF. Human VEGF ELISA antibody did not interact with ovine and equine HCM, indicating that ovine and equine VEGF differ in their amino acid composition from human VEGF. Alignment of human, ovine, equine, and canine VEGF amino acid sequences revealed a high degree of sequence homology (Figure 1).

Although canine VEGF sequences harbour an insertion of 24 amino acids, only two other amino acids (indicated in light blue) differ from the human VEGF sequence. These differences did not interfere with interaction of the human VEGF antibody. However, ovine and equine VEGF differ from human VEGF in four amino acid positions (indicated in yellow or green, respectively). Sequence alteration caused by amino acid exchanges might explain why VEGF of these species cannot be recognized by the human ELISA antibody. Using ovine, equine, and canine VEGF ELISA, we were able to demonstrate the presence and determine the concentrations of VEGF in all HCMs used in this study (Table 1). Since HMGB1 is an important alarmin cytokine produced by cells under hypoxic conditions, we wanted to determine if it is present in HCM and is one of the factors that make HCM such a potent chemoattractive substance for BMSCs. Indeed, high concentrations of HMGB1 (between 8 and 25 ng/ml) were detected in all HCMs (Table 1).

**Table 1:** Concentrations of VEGF-A in human, ovine, equine, and canine HCM determined by ELISA

**Concentration pg/ml**	**Human HCM**	**Ovine HCM**	**Equine HCM**	**Canine HCM**
VEGF-A	1990	1428	40	1960
HMGB1	8077	12747	13676	25151

Control media contained less than 9 pg/ml (human and canine), 30 pg/ml (ovine) or 1 pg/ml (equine) VEGF-A and 2.5 ng/ml (all species) HMBG1.

### Chemoattraction of human, ovine, equine, and canine BMSCs by HCM

**Figure 2:**
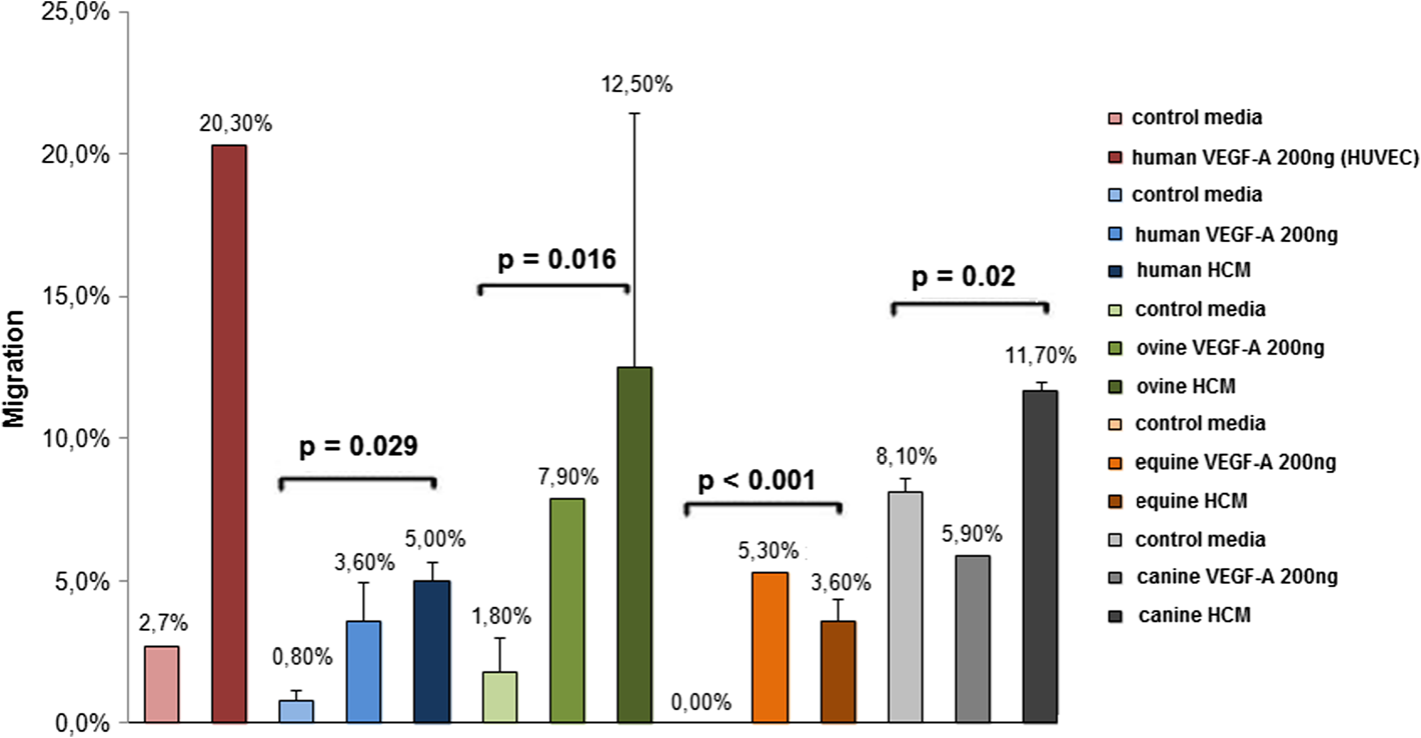
**Migration (in%) of human, ovine, equine, and canine BMSCs towards recombinant VEFG-A or HCM.** HUVECs served as positive control. Differences between migration of BMSCs attracted by control media or HCM were calculated using T test. p values <0.05 were statistically significant.

In order to determine the capacity of HCM to attract BMSCs of human, ovine, equine, and canine origin, we performed migration assays. Human umbilical vein endothelial cells (HUVECs) attracted by recombinant human VEGF (200 ng/ml) served as positive control. As shown in Figure 2, migration of HUVECs increased seven-fold under these conditions. Migration of human BMSCs could be increased 2.36-fold by exposure to 200 ng/ml VEGF. In comparison to control media, human HCM was significantly more potent (p = 0.029). Although human HCM contained only 1.99 ng/ml VEGF (as determined by ELISA), migration of human BMSCs could be enhanced 5.33-fold indicating that HCM contains an additional chemoattractive agent, namely HMGB1. Ovine BMSCs did not respond to human recombinant VEGF (data not shown) indicating that the minor differences detected between human and ovine VEGF (only four amino acids exchanged) were sufficient to result in species specificity. This explains why ovine VEGF in ovine HCM could only be detected by the species-specific ovine VEGF-A ELISA kit. Migration of ovine BMSCs could be increased 5.54-fold by ovine HCM and only 4.3-fold by ovine VEGF (200 ng/ml) (Figure 2). Thus, ovine HCM was significantly more potent than control media (p = 0.016). The same was true of equine BMSCs. Migration could be boosted 3.6-fold when equine HCM was used (p < 0.001). Canine BMSCs migrated less efficiently compared to the other species. However, canine HCM enhanced their migration 1.4-fold (p = 0.02) while canine VEGF was not a potent inducer of migration.

## Discussion

The use of BMSCs in orthopaedic surgery has gained importance over the last years and is rapidly becoming clinical routine. BMSCs are also interesting tools in human and veterinary medicine in general because they are able to migrate and differentiate into osteoblasts in bone tissue [1], they have been proven to regenerate bone combined with carriers [8], and they sustain skin repair in canine animal models by influencing cellular proliferation and angiogenesis, and via modulation of mRNA expression of wound-healing factors [9]. Recently, BMSCs were used successfully in the treatment of horses affected by tendonitis and desmitis [10]. Usually these high performance horses develop skeletal or muscular injuries that are difficult to treat, and after many surgeries they are not able to resume sports activity. Clinical studies have demonstrated that horses treated with equine BMSCs were able to resume previous levels of sport activity [9]. Furthermore, equine autologous cell products are able to stimulate and activate BMSCs facilitating treatment and healing of chronic lesions in these animals [11]. Human BMSCs have been demonstrated to migrate towards chemoattractive agents such as platelet-derived growth factor (PDGF), epidermal growth factor (EGF), and vascular epidermal growth factor (VEGF) [10] as well as towards insulin-like growth factor (IGF-1), interleukin-8 (IL-8), bone-morphogenetic protein (BMP)-4, and BMP-7 [1]. BMSCs are also able to influence repair by secreting growth factors, anti-apoptotic factors, and anti-inflammatory factors [1]. Here, we investigated the directional migration of human, ovine, equine and canine BMSCs towards hypoxia conditioned media (HCM), and found that they contain high concentrations of the potent chemoattractive agent HMGB1, an important alarmin cytokine.

HMGB1 is a non-histone nucleosomal protein which is expressed in all mammal cells, a critical mediator of systemic and local inflammation [7]. It is a chemoattractant, which is directing migration of smooth muscle cells, myoblasts, mesoangioblasts, hematopoietic stem cells, dendritic cells, and human MSCs. Moreover, HMGB1 causes the differentiation of MSCs into osteoblasts [7]. The presence of HMGB1 in HCM explains why they are significantly stronger chemoattractants than pure VEGF.

Our data suggest that HCM have a potent species-specific chemoattractive capacity. Cell migration could be enhanced from 1.4 to 5.54 times depending on the species tested. In comparison to that, pure recombinant VEGF (200 ng/ml) increased migration only from 0 to 4.3-fold. Canine VEGF did not enhance migration of canine BMSCs when compared to control media. However, 8.1% of the canine BMSCs already migrated without any further stimulus in the control media. This could be due to the presence of unknown chemoattracting agents which attract canine BMSCs more efficiently that the other species tested. Nervertheless, canine HCM still boosted migration of canine BMSCs significantly.

In our study HCM contained low concentration of VEGF (from 40- 1990 pg/ml) and high concentrations of HMGB1 (8077-25151 pg/ml). The key for effective migration of BMSCs seems to be the combination of both and other factors present in HCM.

Generation of HCM is easy and cheap compared to preparation and purification of recombinant proteins. Hence, HCM can be applied in large animals (e.g. sheep, horse or dogs) for attraction of BMSCs into tissue defects caused by tumour resection or trauma.

## Conclusions

In this study we show that HCM has a species-specific chemoattractive capacity for human, ovine, equine and canine BMSCs. Generation of HCM is inexpensive and provides biologically active chemoattractive agents without time-consuming and expensive processes of protein enrichment or purification of recombinant proteins. The aim of future studies will be to apply HCM in vivo in large animals to stimulate tissue regeneration by attraction of BMSCs to areas of tissue defects.

## Competing interests

The authors declare that they have no competing interests.

## Authors’ contributions

ARW, AG and MG conceived and designed the experiments; AG and SK performed the experiments. Data were analyzed by AG and SK. AG and RW wrote the paper. All authors read and approved the final manuscript.
